# BRAF V600E-Positive Congenital Multisite Langerhans Cell Histiocytosis

**DOI:** 10.7759/cureus.10200

**Published:** 2020-09-02

**Authors:** Maria Camila Prada Avella, Amaranto Suárez, Sharon Contreras, Alejandra Calderon

**Affiliations:** 1 Pediatric Oncology, Instituto Nacional de Cancerología, Bogota, COL; 2 Pediatric Oncology, Instituto Nacional de Cancerología, Bogotá, COL; 3 Pediatrics, Instituto Nacional de Cancerología, Bogotá, COL

**Keywords:** braf v600e mutation, langerhans cell histiocytosis, congenital langerhans cell histiocytosis, children.

## Abstract

Congenital Langerhans cell histiocytosis (LCH) usually manifests as a disease limited to the skin, with self-healing characteristics; however, in some cases, it may be a more severe entity, with multisystemic expression and poor prognosis. We present the case of a patient diagnosed with multisystemic congenital LCH, with the presence of the BRAF V600E mutation, with a severe form of the disease, with risk organ compromise, and manifestations of resistance to chemotherapy. This case is a challenge due to the disease's biologically aggressive behavior in this patient. It presents unique treatment difficulties as a result of inherent resistance to conventional therapy and uncertain response to BRAF inhibitors.

## Introduction

Langerhans cell histiocytosis (LCH) is a rare entity resulting from the clonal proliferation of cells similar to Langerhans cells (LC) [1,2]. Its incidence is 3 to 5 cases per million [2]. It can appear at any age, but it is more frequent in childhood [1,2]. In the neonatal period, the incidence is one to two cases per million [1,2]. Depending on clinical presentations, it is further distinguished into including Letterer-Siwe disease, Hand-Schüller Christian disease, eosinophilic granuloma, and a congenital self-healing form [3].

LCH is characterized by lesions that include cluster of differentiation (CD)1a + CD207 + dendritic cells and inflammatory cell infiltrates [3]. The molecular analysis clarified that LCH arises from the pathological activation of the mitogenic activity of the protein kinase pathway in myeloid precursors [3]. In particular, the mutation in the BRAF V600E gene encoding serine/threonine-protein kinase BRAF has been identified in ∼50%-60% of patients with LCH [3]. The BRAF mutation has been demonstrated in cases of risk organ involvement, and a statistically significant correlation has also been found between the BRAF mutation and the increase in recurrences in LCH [4].

This disorder can occur at any age and can be completely benign and self-resolving or fatal and refractory [5]. The optimal treatment option for infants with LCH is combination chemotherapy according to the guidelines of the Histiocyte Society 2009 for the evaluation and treatment of LCH [5].

Most cases of congenital LCH involve the skin most of the time and show spontaneous remission [6]; This type of LCH is known as "self-healing or self-healing congenital LCH" or "Hashimoto-Pritzker disease" [6]. Cases of congenital multisystem-type LCH have rarely been reported, and most of them were fetal [6].

Congenital LCHs that are limited to skin lesions are generally thought to be clinically benign, with a good prognosis, but cases of unusual presentation have a poor outcome [7].

## Case presentation

**Figure 1:**
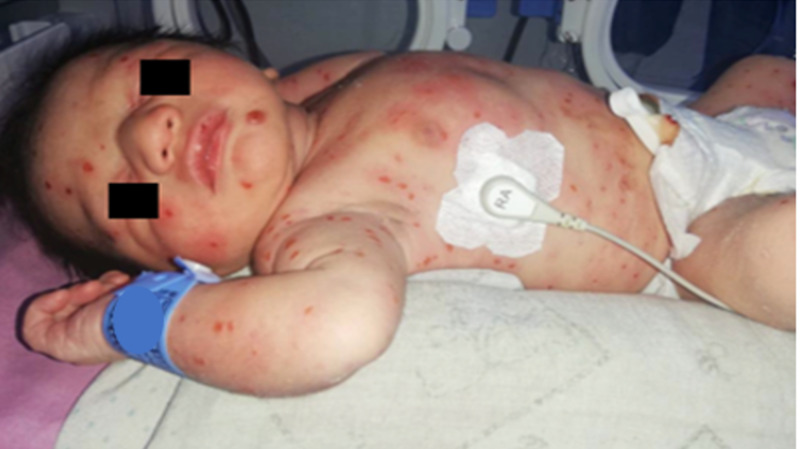
Disseminated erythematous plaques and papules

A 4-month-old male, with no significant medical history, upon birth presented papular, erythematous lesions on approximately 90% of the body surface, later turned into crusty, painful lesions, some bleeding, which disappeared with finger pressure. Initially, he consulted a medical center where they took a skin biopsy with findings suggestive of histiocytosis (Figure 1).

Physical examination revealed crusted hyperemic lesions on the soft and hard palates, no masses or hepatosplenomegaly were palpable in the abdomen, there were widespread scattered lesions of the skin of varying size, erythematous, some dry with crusts, others with little bleeding suggestive of cell histiocytosis from Langerhans. Rest of the physical examination without relevant findings. The admission exams showed a hemoglobin of 9.8 mg/dl, platelets 32 × 109/L, fibrinogen 57 gr/dl, prothrombin time (PT) 22.1 seconds (control 13 seconds), partial thromboplastin time (PTT) 34.7 seconds (control 31.2 seconds), preserved kidney and liver function, normal electrolytes, negative serology results for hepatitis B and C, human immunodeficiency virus (HIV), Epstein Barr virus (EBV) and human cytomegalovirus (HCMV).

**Figure 2:**
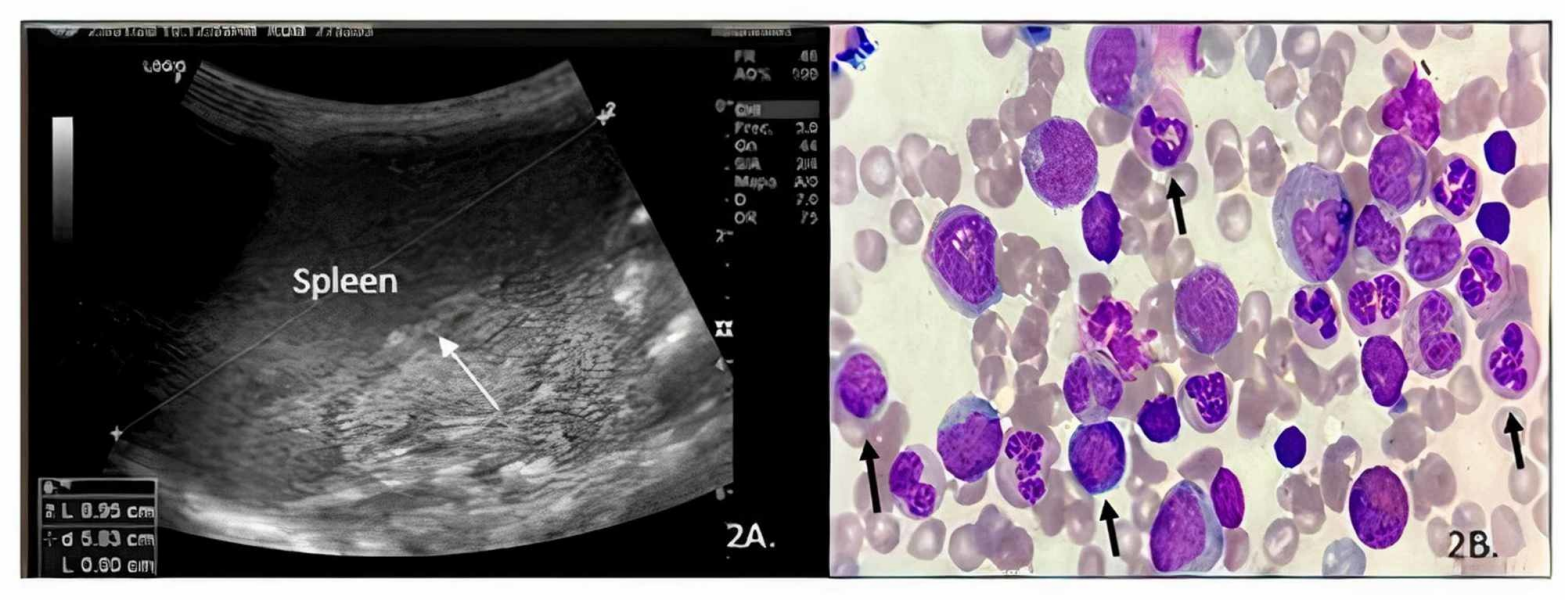
A. Ultrasound of the abdomen that showed splenomegaly; B. Myelogram was hypocellular with mainly granulocytic and erythroid hematopoiesis

Extension studies were performed, an ultrasound of the abdomen showed splenomegaly (Figure 2A). In the bone series, no bone or soft tissue lesions were detected, the chest radiograph was normal, echocardiogram without alterations, the peripheral blood smear showed slight anisocytosis with macrocytes and microcytes. The myelogram was hypocellular with mainly granulocytic and erythroid hematopoiesis (Figure 2B).

**Figure 3:**
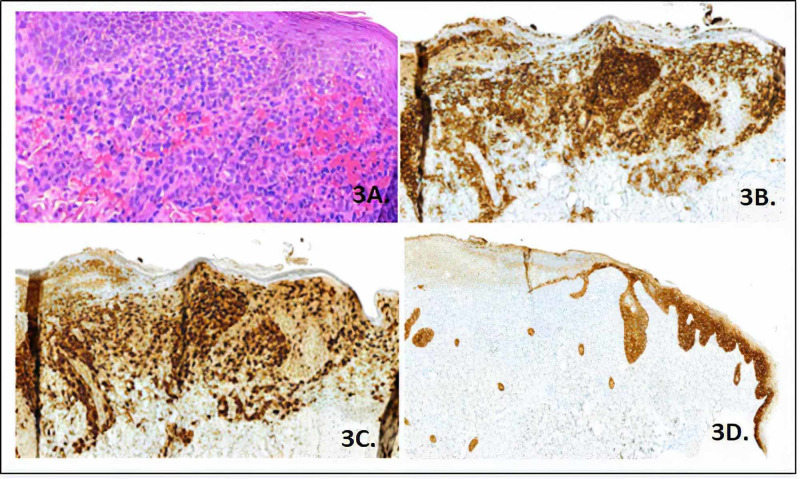
Immunohistochemistry A. Skin biopsy specimen from the lesion: Hematoxylin-eosin stain x40. Epidermis inflammatory cells with erosion and inflammatory costra, massive eritrocitary extravasation, and mature lymphocytes in lower proportion. In the surface dermis is a massive occupation of some intermedie sites cells, histiocytes had folded nuclei and acidophilic cytoplasm, and some kidney-shaped nuclei; B. CD1a immunohistochemical staining x200; C. S100 immunohistochemical staining x200; D. Cytokeratin 5/6 immunohistochemical staining x200.

Skin biopsy revealed some attenuation by exocytosis of inflammatory cells with erosion and inflammatory scab on the epidermis, strong reactivity of the positive cells for CD68, protein S100, CD1a, and langerin, a low and reactive CD3 T lymphoid population and negative CD20. Cytokeratin 5/6 highlighted attenuation and erosion of the epidermis in some areas by the massive infiltration of positive cells. Compromise by histiocytosis of LC was considered (Figure 3).

The bone marrow aspirate sample was cytogenetically performed, the conventional karyotype was normal, and the BRAF V600E mutation was detected.

With the diagnosis of LCH with a multisystem and risk organ compromise, it was decided to start chemotherapeutic management with a prednisone and vinblastine scheme.

The evaluation at the end of the first chemotherapy cycle showed anemia (hemoglobin 9 mg/dl), the chest radiograph was normal, and hepatosplenomegaly was documented in the total abdomen ultrasound, the bone marrow biopsy without histiocytosis.

**Figure 4:**
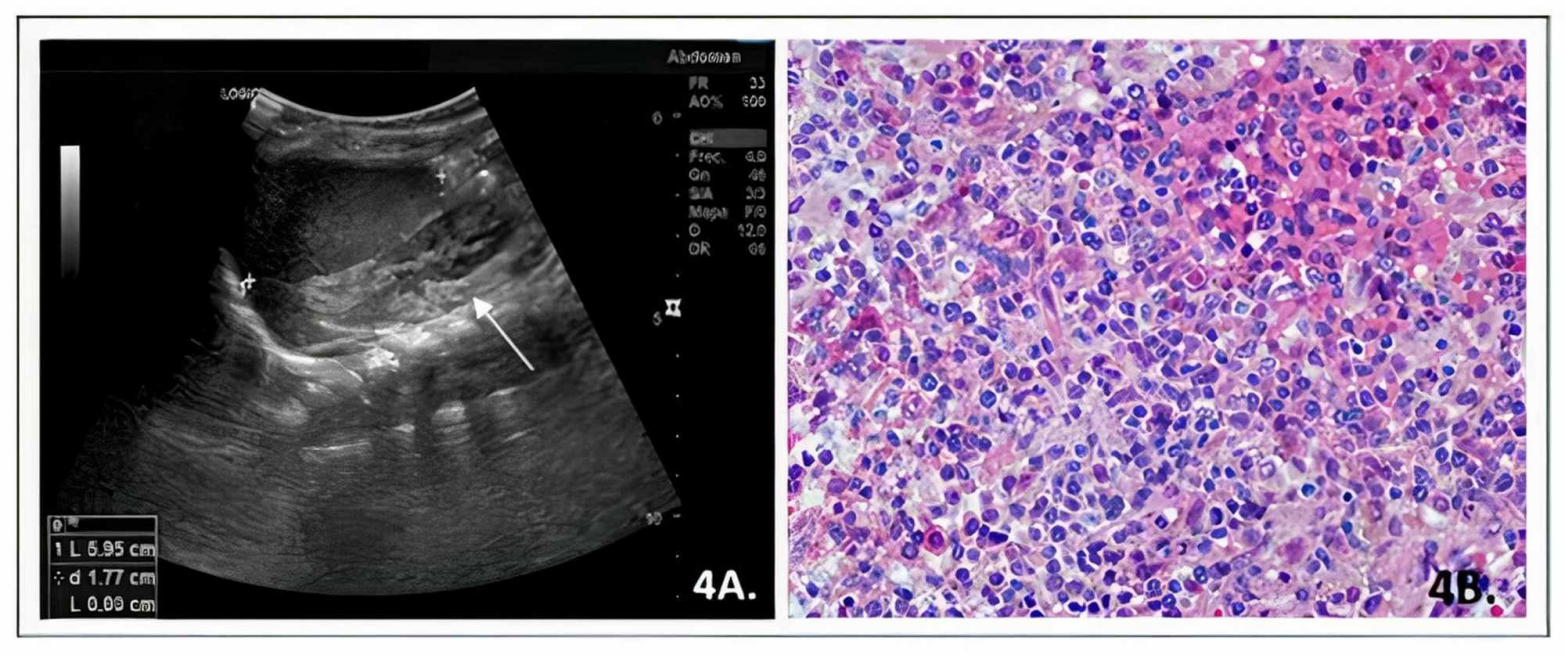
A. Total abdominal ultrasound with a slight parenchymal liver disease without hepatomegaly or splenomegaly; B. Bone marrow biopsy with infiltration by Langerhans cell histiocytosis

It was considered that it was a refractory histiocytosis, so it was decided to administer a second line of treatment, with a regimen based on cladribine and cytarabine. Studies after two cycles of this regimen showed persistent anemia, normal liver and kidney function, total abdominal ultrasound with a slight parenchymal liver disease without hepatomegaly or splenomegaly, and bone marrow biopsy with infiltration by LCH (Figure 4).

Given the partial response to the second line of chemotherapy, the administration of a third cycle was indicated.

## Discussion

LCH is an inflammatory neoplasm of myeloid origin characterized by the classic presence of CD1a +/CD207 + cells [3]. A debate on LCH clustering was finally resolved in favor of neoplasia after discovering the BRAF V600E mutation in 2010 [8-10]. Pathological cells in this entity are involved in near-universal activation of the mitogen-activated protein kinase (MAPK)/extracellular signal-regulated kinase (ERK) pathway, with identified mutations in most ERK kinases (RAS/RAF/MEK) [8-10].

The clinical behavior of LCH is heterogeneous, with cases of self-healing disease, and other cases of greater aggressiveness, with an inadequate response and refractory treatment, like in the case described herein an infant with the BRAF V600E mutation in whom LCH lesions were present from birth, with aggressive behavior of the disease.

In 2016, Héritier et al. [9] published a study that analyzed 315 patients with LCH, of which 173 (54.6%) had the BRAF V600E mutation; these patients showed greater severity of the disease than patients with wild-type BRAF [9]. Patients with the BRAF V600E mutation were 87.8% of the cases with a multisystemic disease with a risk organ (P < .001), P < .002 was considered statistically significant [9]. In the same study, cases with the BRAF V600E mutation were associated with risk organ compromise that could lead to permanent and irreversible damage to the nervous system (75%) and pituitary (72.9%) [9].

Regarding treatment sensitivity, BRAF V600E patients showed resistance to vinblastine and steroids, a high rate of reactivation of the disease, and a higher risk of sequelae [9,11].

Our patient presented a multisystemic form of the disease and with risk organ compromise, with resistance to the first line of chemotherapy treatment, requiring the administration of the second line of chemotherapy with cladribine and cytarabine, also with inadequate response. Resistance to chemotherapy in patients with LCH with the BRAF V600E mutation raises the possibility of treating this group with BRAF inhibitors. In a study published in 2019, Ruan et al. [12], reported that dabrafenib was effective and well-tolerated in the group of LCH with cerebral parenchymal compromise, however, in some of the patients in whom a dose reduction was made, disease progression occurred, so it is necessary to adequately monitor the patients in whom implement this therapy and decide to make adjustments. The use of systemic chemotherapy for cases of multisystemic LCH has improved the prognosis [13]; however, the mortality rate of patients with risk organ involvement remains high, ranging from 16% to 38% [13].

## Conclusions

Congenital LCH is a rare disorder. The clinical presentation of LCH is heterogeneous, as practically every organ or system may be affected. This disorder can be completely benign and self-healing or fatal and refractory. Patients with BRAF V600E mutation have shown greater severity of the disease than patients with wild-type BRAF. Patients with BRAF V600E mutations have resistance to chemotherapeutic treatment, a high rate of reactivation of the disease, and an increased risk of sequelae. The current challenge is to implement this biological discovery as a clinical tool to find better outcomes for children and adults with LCH.

## References

[1] Frade AN, Godinho MM, Batalha ABW, Bueno APS (2017). Congenital Langerhans cell histiocytosis: a good prognosis disease?. An Bras Dermatol.

[2] McKenzie S, Vecerek N, Kang Y, Knowles B, Hogeling M (2019). A neonatal pustule: Langerhans cell histiocytosis. Dermatol Online J.

[3] Pan Y, Zeng X, Ge J, Liu X, Chen Y, Zhou D (2019). Congenital self-healing langerhans cell histiocytosis: clinical and pathological characteristics. Int J Clin Exp Pathol.

[4] Nann D, Schneckenburger P, Steinhilber J (2019). Pediatric Langerhans cell histiocytosis: the impact of mutational profile on clinical progression and late sequelae. Ann Hematol.

[5] Singh A, Mandal A, Singh L, Mishra S, Patel A (2017). Delayed treatment response in a neonate with multisystem Langerhans cell histiocytosis: case report and review of literature. Sultan Qaboos Univ Med J.

[6] Tamefusaa K, Ishidab H, Washioa K, Ishida T, Morita H, Shimada A (2019). Remission of congenital multi-system type Langerhans cell histiocytosis with chemotherapy. Acta Med Okayama.

[7] Inoue M, Tomita Y, Egawa T, Ioroi T, Kugo M, Imashuku S (2016). A fatal case of congenital Langerhans cell histiocytosis with disseminated cutaneous lesions in a premature neonate. Case Rep Pediatr.

[8] Thacker NH, Abla O (2019). Pediatric Langerhans cell histiocytosis: state of the science and future directions. Clin Adv Hematol Oncol.

[9] Héritier S, Emile JF, Barkaoui MA (2016). BRAF mutation correlates with high-risk Langerhans cell histiocytosis and increased resistance to first-line therapy. J Clin Oncol.

[10] Abla O, Weitzman S (2015). Treatment of Langerhans cell histiocytosis: role of BRAF/MAPK inhibition. Hematology Am Soc Hematol Educ Program.

[11] Awada G, Seremet T, Fostier K, Everaert H, Neyns B (2018). Long-term disease control of Langerhans cell histiocytosis using combined BRAF and MEK inhibition. Blood Adv.

[12] Ruan G, Goyal G, Abeykoon J (2019). Low-dose BRAF-inhibitors in the treatment of histiocytic disorders with the BRAF-v600e mutation. Blood.

[13] Morimoto A, Oh Y, Shioda Y, Kudo K, Imamura T (2014). Recent advances in Langerhans cell histiocitosis. Pediatr Int.

